# Editorial comment to “Impact of COVID‐19 infection on the in‐hospital outcome of patients hospitalized for heart failure with comorbid atrial fibrillation: Insight from National Inpatient Sample (NIS) database 2020”

**DOI:** 10.1002/joa3.13093

**Published:** 2024-06-06

**Authors:** Yasushi Mukai

**Affiliations:** ^1^ Division of Cardiology Fukuoka Red Cross Hospital Fukuoka Japan

Editorial comment to “Impact of COVID‐19 infection on the in‐hospital outcome of patients hospitalized for heart failure with comorbid atrial fibrillation: Insight from National Inpatient Sample (NIS) database 2020” by Wattanachayakul P, et al.^1^

Numerous clinical studies have continuously reported the increased incidence and worse clinical outcomes of cardiovascular diseases associated with COVID‐19. Relevant cardiovascular diseases include myocarditis, acute coronary syndrome, heart failure (HF), thromboembolisms, and arrhythmias.^2^ It is also important to note that having COVID‐19 results in more complicated clinical courses and higher mortalities in patients with preexisting cardiac conditions.^3^ The present study by Wattanachayakul et al. utilized a big database of the US Healthcare systems and revealed a strong relation between COVID‐19 infection and adverse outcomes in hospitalized HF patients with atrial fibrillation (AF).

## 1 RELATIONSHIP OF HF/AF TO COVID‐19 AND ASSOCIATED RISK

A number of retrospective studies reported more severe conditions and a higher mortality among HF patients with COVID‐19, and that HF was an independent risk factor for acute circulatory failure, renal failure, and multiorgan failure in patients with COVID‐19.^2^ It was also reported that COVID‐19 infection is associated with an increasing incidence of atrial fibrillation.^4^ A preexisting AF is associated with an increased mortality of over twofold in COVID‐19 Patients.^5^ From another aspect, the present study demonstrated that hospitalized HF patients with AF and COVID‐19 had over threefold higher in‐hospital mortality compared with those without COVID‐19. More adverse outcomes such as prolonged length of stay or mechanical ventilation in the studied patients with COVID‐19 were also striking. Whereas COVID‐19 itself can elicit critical conditions, it is also conceivable that COVID‐19 induces or even exacerbates HF and/or AF, which result in adverse clinical outcomes.

## 2 MECHANISMS OF AF OCCURRENCE AND EXACERBATION IN COVID‐19

Cardiovascular involvement of COVID‐19 can be largely explained by its inflammatory mechanisms called cytokine storm, myocardial damage, and relevant endothelial dysfunction.^2^ Adverse effects of COVID‐19 on cardiac function may also include an increased adrenergic drive because of fever and hypoxemia, which increases myocardial damage along with cardiomyocyte infection and cytokine storm. An increased inflammatory response is also related to the occurrence of AF.^2^, ^4^ Indeed, atrial electrical instability and atrial tissue remodeling could be elicited in relation to various cytokine signaling. In addition to systemic inflammation, local mechanisms that contribute to atrial electrical instability associated with COVID‐19 have been considered.^4^ Angiotensin‐converting enzyme‐2 (ACE‐2) has been identified as a functional receptor at cellular membrane for coronaviruses. ACE‐2 is expressed in pneumocytes, macrophages, endothelial cells, pericytes, and cardiomyocytes and thus should be ubiquitously expressed in the atrial wall. In a normal condition, ACE‐2 acts as a suppressor of angiotensin II (AngII)‐induced cellular signaling. Ang II‐induced signaling lead to myocyte hypertrophy, vasoconstriction, tissue fibrosis, and an increased oxidative stress, most of which is associated with AF occurrence and persistence. Binding of ACE‐2 with SARS‐CoV‐2 results in a functional reduction of ACE‐2 at the cellular membrane, which may augment Ang II‐induced signaling. Such local mechanisms may be involved in the occurrence and exacerbation of AF and adverse clinical outcomes in patients with cardiovascular diseases and COVID‐19.

## 3 FUTURE PERSPECTIVE

Adverse effects of COVID‐19 on the cardiovascular system exert a wide range of morbidity including AF and are associated with worse clinical outcomes. Future investigations are needed to further understand how to manage such clinically difficult conditions.

## CONFLICT OF INTEREST STATEMENT

The authors declare no conflict of interests for this article.
